# Insulation for Daydreams: A Role for Tonic Norepinephrine in the Facilitation of Internally Guided Thought

**DOI:** 10.1371/journal.pone.0033706

**Published:** 2012-04-06

**Authors:** Jonathan Smallwood, Kevin S. Brown, Benjamin Baird, Michael D. Mrazek, Michael S. Franklin, Jonathan W. Schooler

**Affiliations:** 1 Department of Social Neuroscience, Max Planck Institute for Human Cognitive Brain Sciences, Leipzig, Germany; 2 Department of Physics, University of California Santa Barbara, Santa Barbara, California, United States of America; 3 Department of Psychological and Brain Sciences, University of California Santa Barbara, Santa Barbara, California, United States of America; University of California, Davis, United States of America

## Abstract

Although consciousness can be brought to bear on both perceptual and internally generated information, little is known about how these different cognitive modes are coordinated. Here we show that between-participant variance in thoughts unrelated to the task being performed (known as task unrelated thought, TUT) is associated with longer response times (RT) when target presentation occurs during periods when baseline Pupil Diameter (PD) is increased. As behavioral interference due to high baseline PD can reflect increased tonic activity in the norepinephrine system (NE), these results might implicate high tonic NE activity in the facilitation of TUTs. Based on these findings, it is hypothesised that high tonic mode NE leads to a generalised de-amplification of task relevant information that prioritses internally generated thought and insulates it from the potentially disruptive events taking place in the external environment.

## Introduction

A characteristic of consciouness is the ability to focus on information that is independent of the events occurring in the external world [1]. Just as attention to sensory data aids external goal achievement [2], freedom from perceptual input facilitates abstract mental capacities such as creativity, logical analysis, and planning [3]. To coordinate these different modes of thought, processing resources must be allocated appropriately in order to attain the favored internally or externally oriented cognitive state. For example, when the external environment is salient, such as performing an unfamiliar task [4], [5] or a task that depends directly on current environmental information [6], attention is generally directed to perception and imaginative thoughts are infrequent and often less structured [5]. Conversely, because during internally motivated thought mental content does not depend on external information, when the mind wanders from the task being peformed concurrent environmental input is often neglected [7]. The attentuation of external information that occurs when consciousness turns inwards is known as perceptual decoupling [8] and is hypothesised to be adaptive because it reduces attention to unrelated external events that could otherwise derail the internal train of thought [9]. Given that distinct configurations of information processing resources are required for internal and external cognitive states [8], [10], [11], [12] the identification of the neural mechanism(s) that govern the tradeoffs between these different modes of thought is an important question in understanding how conscious thought operates.

The norepinephrine (NE) system, particularly the brainstem locus coeruleus (LC), has a well-defined role in dynamic, goal-directed behavior, in part due to the multi-phasic nature of the system's activity. Studies suggest that the NE system exhibits three distinct firing modes each of which serve to promote categorically unique behavioral outcomes [13], [14]: (i) low baseline levels of LC activity are associated with drowsiness or somnambulance, supporting a state of inactivity, (ii) transient bursts of LC activity synchronized to task events maximize the signal to noise ratio (SNR) of task-relevant information which help to maintain attention on the current goal, and (iii) high baseline, or tonic, LC activity reduces the SNR of task relevant stimuli leading to poor task performance. In animals, the high tonic NE state often entails a hyper-sensitivity to external stimuli, although one recent hypothesis of the tonic NE state in humans and non-human primates is that the reduction in the SNR of external task relevant information may support disengagement from the current goal, which allows for an active mental exploration of alternative possibilities [13], [14].

Central to this Adaptive Gain Theory (AGT) is the notion that LC activity acts to increase the gain on cortical processes and that variations in the temporal coupling between activity in this system and events in the current task can bias cognition towards or away from external task relevant information [13], [14]. According to AGT, the LC-NE system leads to a generalised amplification of cortical processing. When LC activity is coupled to task relevant events (the *phasic* mode) this system acts like a temporal filter: the correspondence in time between NE release and the onset of cognitive processing of task relevant events emphasises cortical representations of information related to the task being performed. By contrast, when the baseline firing rate of the LC is high (the high *tonic* mode), NE release is *uncoupled* from events in the task. In the high tonic mode, the absence of the temporal coupling between LC activity and task events leads to an undifferentiated increase in cortical processing and because NE release no longer prioritises task relevant representations it can be conceived of as a net amplification of informational content unrelated to the task in hand. By boosting task-irrelevant signals in this manner, the high tonic mode of the NE system could facilitate the conscious expression of internal thoughts that are not directly related to the current task.

Although increasing cognitions that are irrelevant to the task in hand has a detrimental impact on immediate performance, it can be adaptive in a situation when the task has low incentive value. In low incentive tasks, a de-prioritization of task-relevant information allows alternative behaviors to be mentally explored, which in turn could potentiate the pursuit of goals with a greater long-term payoff [12], [13]. Humans frequently think about things other than the task that they are performing in both the laboratory [11] and in daily life [15], [16], especially when thask has low incentive value, and these thoughts often take the forms of plans or goals for the future [17]. Cognition focused on the evaluation of goals other than those posed by the current task are an example of an exploratory goal state. Evidence for a role of the LC system in exploratory control states comes from studies linking NE release to shifts in strategy during the performance of gambling tasks [16]. We hypothesized that under relatively benign circumstances conditions, task unrelated thought (TUT) could represent an example of an exploratory control state and so would be facilitated by the the capacity for high tonic LC activity to increase the gain on task irrelevant cognitive representations.

To examine this question, we analyzed the data from a sample of 19 participants who performed a simple vigilance task while Pupil Diameter (PD) was measured. Although PD is controlled by a circuit that includes areas in the brain and the periphery other than the LC, it is often used as a proxy for NE system activity because (i) in nonhuman primates LC activity is associated with increases in baseline PD [13], (ii) human PD shows a Yerkes-Dodson relationship to performance that is predicted by accounts of NE function [18], [19], [20], [21], and (iii) our previous work has demonstrated that PD exhibits tonic and phasic dynamics that co-vary with how the different modes of NE functioning may relate to task engagement [22]. After completing the task participants completed a commonly used self-report measure of the TUT they experienced during the experiment [9], [23]. Examples of the TUT measured by this questionnaire include thoughts regarding episodes from the past, anticipations of events that may take place in the future, as well as thoughts regarding family and friends.

Our hypothesis was that elevations in baseline PD coupled with performance decrements would indicate that the constraints placed on attention by the current task have been reduced, facilitating the experience of TUT. Based on previous research [18], [19], [20], [21] we expected to observe the classic Yekes-Dodson pattern linking both large and small PD to performance decrements, which we quantify as longer reaction time (RT) to targets. Although very low and very high levels of LC activity are detrimental to externally driven task performance, if the high tonic mode of the NE system facilitates the experience of TUT, the variance linking very **large** PD to **slower** RT should increase with individual differences in TUT. To test this hypothesis we examined (i) if the expected quadratic relationship between PD and RT becomes linear when between participant variance in TUT is taken into account and (ii) whether between participant variance in TUTs was associated with the longer RT that occurs when PD is large (but not small).

## Methods

This study was granted ethical approval by the University of California, Santa Barbara Ethics Committee (Code 09306) and written informed consent was acquired from every participant prior to participation. Pupil size and gaze direction were acquired using a Tobii 120 eye tracker (Tobii, Stockholm, Sweden) with a sampling rate of 125 Hz. Participants (9 Males, Mean Age = 19.5, SE = .26) were seated on a comfortable chair, approximately half a meter from the eye tracker. Prior to data collection the eye tracker was calibrated to each individual. For each PD sample collected, the Tobii automatically assigns a measure of quality to the sample. These values can be either “good” (1) or “bad” (0). This quality measure is assigned separately for each eye: therefore either, one, or both eyes can be flagged as “good.” We employed this measure in our data processing pipeline as follows. If the Tobii assigned a “good” rating to measurements from both eyes the two pupil diameters were averaged. If only one eye was flagged as “good” that measurement was used for PD at that time point. Any remaining times in which both eyes were flagged as “bad” were interpolated; we used basic linear interpolation, which fills in the gaps using neighboring “good” PD values. These interpolated gaps were generally short (due to either blinks or the hooding of the eye by eyelashes), and participants were discarded if their resulting PD time series consisted of more than 50% interpolated data (n = 15). Our data processsing steps and proportion of participants excluded due to poor data are consistent with our previous investigation [21]. The data was then median filtered (order 5) in order to remove spikes and subsequently low-pass filtered with a cutoff frequency of 10 Hz.

Participants performed a single twenty minute session of a vigilance task in which they were asked to detect targets (the letter X) presented in a sequence of nontarget numbers (the digits 1–9). Stimuli were presented for 1000 milliseconds with jittered fixation delays that varied between 2000 and 2500 milliseconds. Stimuli were quasi-randomized so that target occurrence was unpredictable. Participants received an average of 39 targets (range: 34 to 43). Mean correct response time was 492 msecs [SD = 168] and accuracy was high [Mean = .95 (SD = .22)].

Immediately after completion of the task TUT was measured using the Dundee Stress State Questionnaire [22], [23]. The DSSQ consists of two subscales: Task Related Interference (TRI) measures interfering thoughts about how the task was performed (such as “I thought about how poorly I performed”) and (ii) TUT assesses internally generated thoughts (such as “I thought about an event in the recent past”, “I thought about something that might happen in the near future”). Participants rate their agreement with each question on a scale of 1 to 5, and subscale averages (both TUT and TRI) were calculated for each individual.

## Results

**Figure 1:**
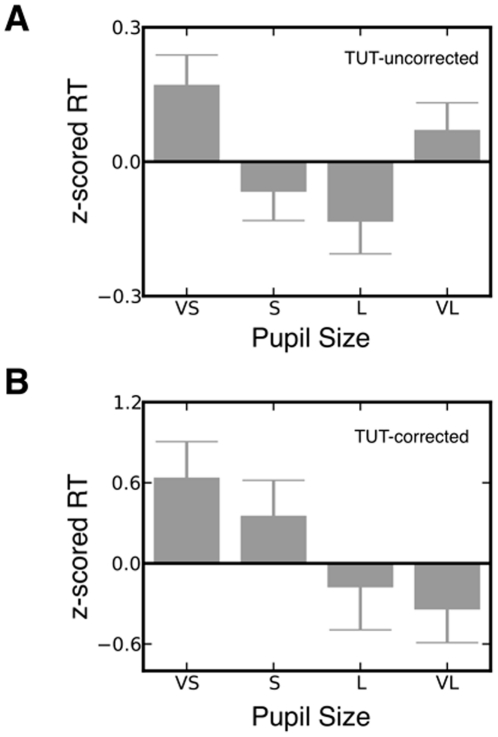
Relation between pupil diameter, response time and task unrelated thought. **A** Group-average standardized RT, for four pupil diameter quartiles (VS, very small; S, small; L, large; VL, very large), showing a classical U-shaped relationship between PD and RT. The error bars indicate one standard error of the mean. **B** Group-average parameter estimates for the impact of PD and RT when the impact of TUT has been partialed out. It is clear that after the experience of TUT has been accounted for in the model, the impact of PD on RT is linear rather than quadratic.

After processing PD was averaged at the group level into four equally sized bins of increasing size based on the aggregate cumulative distribution. Within-subject z-scored RTs were averaged according to the appropriate pre-target PD bin (see Figure 1A) and a repeated measures Analysis of Variance (ANOVA) confirmed a significant quadratic relationship between PD and RT [*F*(1,18) = 7.70, *p*<.01, *η^2^* = .30]. Replicating previous studies [18], [19], [20], [21] planned one tailed t-tests indicated that relative to large pupils, pupils with very small (p<.01) and very large diameters (p<.03) were detrimental to performance as indexed by longer RT. To explicitly test whether TUT accounted for the RT variance associated with large (but not small) PD, the omnibus ANOVA was repeated including z-scored TUT as a continuous regressor. This altered the relation between PD and RT from a quadratic to a linear pattern [*F*(1,17) = 6.23, *p*<.05, *η^2^* = .24] indicating that the parabolic feature of the initial relationship between PD and RT was in part associated with TUT. To represent the effect of PD on RT when the impact of TUT had been controlled for we calculated the parameter estimates for each of the four pupil dilation bins for the GLM when TUT was included (see Figure 1B). Without controlling for TUT there was no reliable difference between very large and very small pupils (p>.3). However, when individual differences in TUT were controlled RT associated with very small pupils was longer than that associated with very large pupils (p<.05). Thus, once TUT was taken into account, very large PD was associated with shorter RT than was very small PD. The lengthening of RT that was observed when targets were presented during periods with large baseline PD was, therefore, correlated with individual variation in the experience of TUT.

**Figure 2:**
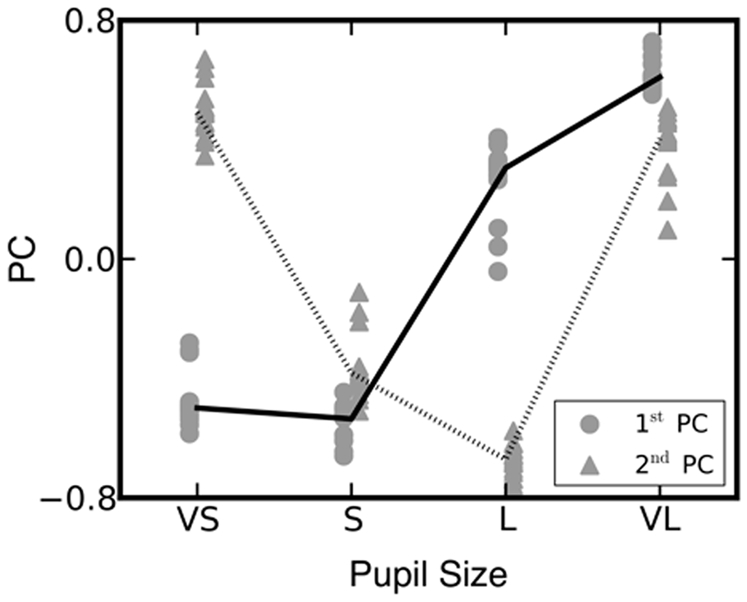
The first two principal components (PCs) of the subject-level relation between pupil diameter and RT. The solid and dotted lines respectively show the first and second PCs from the full (19-subject) data matrix. The filled circles and triangles show these same components for 19 jackknife samples in which each subject in turn was left out of the PC analysis.

The omnibus ANOVA also revealed a linear PD X TUT interaction [*F*(1,17) = 5.3, *p*<.05, *η^2^* = .24] indicating that between participant variance in TUT was significantly correlated with the relationship between PD and subsequent RT. To understand whether this relationship supported the hypothesis that the performance decrements associated with high tonic mode of NE were greater when individuals report high levels of TUT the PD RT data were decomposed at the group level using principal components analysis. This analysis allows different types of relationship between PD and RT to be captured by a series of discrete variables which can then be related to between participant variation in TUT. This analysis revealed two components that accounted for approximately 78% of the observed variance (see the inset of Figure 2 and Table 1). In order to obtain error bars on the correlations reported between subject scores from the first two principal components (PCs) and TUT and TRI, we used a subject-level jackknife analysis. The PC decomposition was repeated nineteen times, with the PD/RT data for each subject left out in turn. Resampling analyses that involve principal component decompositions can be difficult for two reasons: PCs in the resampled data may have signs reversed (with the corresponding scores reversing as well in order to properly reconstruct the signs in the data matrix) and, if not originally well separated in terms of explained variance, may swap identity. No identity switches were observed in our jackknife samples; to correct for sign reversals, we took the PCs arising from the decomposition of the full data matrix as the “canonical” signs. Each resampled set of PCs was correlated with its corresponding canonical PC; if that correlation was negative, the sign on both the resampled PC and the scores were reversed. Table 1 shows the results from the jackknife.

**Table 1:** Results from the subject-level jackknife on the principal component decomposition of the pupil diameter/reaction time matrix.

Component	Fraction of Variance Explained	Correlation with TUT	Correlation with TRI
1	0.44 (14)	0.49 (32)	0.09 (48)
2	0.34 (12)	0.06 (76)	−0.19 (54)
3	0.20 (11)	-	-
4	0.03 (2)	-	-

Numbers in parentheses are two jackknife standard deviations.

**Figure 3:**
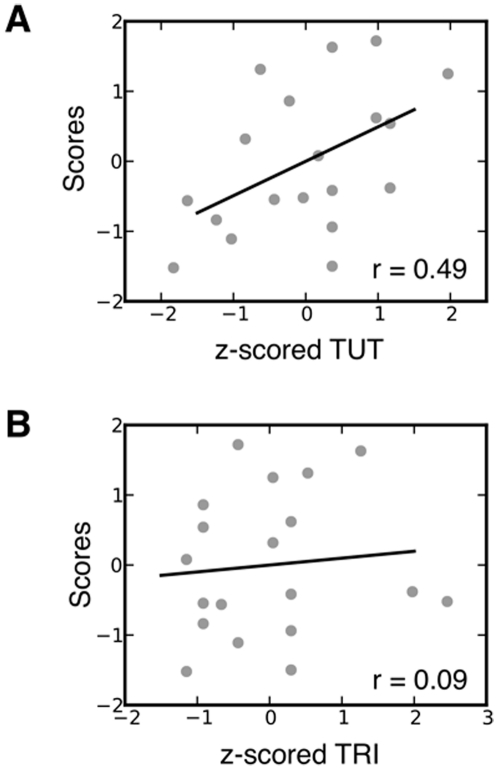
The group level association between the first principal component of PD and RT and the experience of TUT. **A** TUT has a correlation with subject scores from the first PC that is statistically different from zero (r = 0.49, two jackknife SD 0.32). **B** TRI does not correlate with subject scores from the first PC (r = 0.09, two jackknife SD 0.48).

Component 1 represents a linear relationship such that individuals who tend to weight positively on this component tend to show longer RT as PD increases, while a negative weighting on this component indicates that generally RT lengthens as PD decreases. Component 2 has a clear parabolic form and so reflects a u-shaped relationship between PD and RT. Analysis of the correlations between the loadings of these two components and self reports of TUT indicated that only the positive association with the first component [*r* = 0.49 (two jackknife SD 0.32)]^i^ was statistically different from no correlation (see Figure 3A). The association between thoughts unrelated to the current task and the first PD/RT component indicates that as TUT increases across individuals within our sample, increasing baseline levels of PD prior to target presentation are increasingly likely to lead to slower RT.

Although the association between PD and RT is consistent with the hypothesis that one consequence of high tonic NE is to bias attention away from the task and so facilitate thoughts unrelated to current environmental input, it could also be explained by supposing that any form of internally maintained cognitive experience would manifest as longer RT associated with large PD. To examine this alternative hypothesis we explored how TRI influenced the PD/RT link. Correlations between individual subject TRI and PD/RT components were not significantly different from zero (see Figure 3B), indicating that the relationship between large pupils and long RTs is a specific marker for conscious thoughts that depend on content unrelated to the current task; thoughts re-appraising current task performance play no role in the PD/RT relationship.

## Discussion

This experiment demonstrated that between-subject variance in TUT accounted for a significant portion of the RT cost that was incurrred when targets were presented in a period when pupils were dilated, a result that corroborates previous observations that link elevations in tonic PD with cognitions that are not related to the current task. Previous studies have demonstrated that both baseline PD and TUT increase in tasks when external events have minimal task value (Experiment 1, [21]) and that spontaneous elevations in baseline PD occur when task events in the external environment are not encoded properly or when the retrieval of task relevant information from memory takes longer (Experiments 2 and 3, [21]). Together such results indicate that elevations in baseline PD co-vary with situations when attention is less firmly directed to the task in hand and under which TUT is likely to occur.

At a psychological level, these data inform our understanding of the information processing features that must be accounted for when explaining the capacity to engage in TUT. Based on theses results, engaging in TUT does not simply entail a state of heightened perceptual sensitivity/external distraction. In this experiment, there was no association between longer RT, higher PD and distracting thoughts regarding the task (see Figure 3B) and the same measure of TUT was previously associated with a reduced processing of distracters during signal detection (e.g. [23]). Moreover, although states of somnambulence or drowsiness also reduce attention to perception, these data suggest that TUT is not simply a corollary of underarousal. In the current experiment, TUTs were linked to behavioral decrements when PD was larger rather than smaller, indicating higher rather than lower arousal, and this observation fits with previous studies demonstrating increases in other peripheral measures such as heart rate TUT [24]. Taken together with previous investigations the current data suggest two distinct psychological features are necessary to explain the experience of TUT: (i) relatively high tonic levels of baseline arousal that may reflect internally generated cognitive operations and (ii) a reduced sensitivity to events in the environment (known as perceptual decoupling) that reduces attenton to task and may serve to insulate the internal train of thought from external disruption (see [21] for a discussion).

As PD is increasingly used as a peripheral indicator of the function of the NE system these data also provide provisional confirmation for the hypothesis that the NE system can facilitate TUT. Confidence that the high tonic NE mode may be linked to TUT is strengthened by a number of empirical features that both states share. Impaired response inhibition is often linked to TUT [25], [26] and can be alleviated by a pharmacological alteration of tonic NE levels [27]. Likewise, the P3 event related potential which indexes task relevant attention and memory [28] is diminshed by increased tonic LC activity [29] and has a reduced amplitude during TUT. [7], [9], [30] These observations provide support for the hypothesis that high levels of tonic NE facilitate internal thought that does not rely on PD as an indicator of LC activity. At a more general because thinking about future autobiographical goals makes up a large proportion of TUT [17] these results provide a novel source of evidence for the AGT hypothesis that high tonic NE optimizes exploratory control states [12], [13].

Although evidence for an association between TUT and the tonic mode of the NE system does not depend solely upon the validity of PD as an indicator for LC function, PD is influenced by both central and peripheral nervous system sites other than the LC. Critically despite evidence of a correlation between LC activity and PD, there is no agreed upon mechanism for how the former influences the latter. This has led to the suggestion that both LC and PD receive input from a comon source, for example the paragigantocellularis nucleus of the ventral medulla (e.g. [29]). Such issues notwithstanding, given that TUT occupies almost half of daily life [15], [16] this experience may provide a novel source of evidence with which to understand the role of the LC-NE system in co-ordinating cognition. Future work could profit from the use of more direct measures of LC activity, or methods to manipulate NE function, to explore whether this system facilitates a decoupling of attention from the external environment that facilitates internal thought and insulates the daydreaming mind against the distractions of the outside world.
